# Infective Endocarditis Following Coil Embolization for a Visceral Pseudoaneurysm: A Case Report

**DOI:** 10.7759/cureus.89484

**Published:** 2025-08-06

**Authors:** Matthew Kaldas, Sofia Gonzalez Rubiano, Benjamin Pendowski, John Greene, Bela Kis

**Affiliations:** 1 Internal Medicine, Nova Southeastern University Dr. Kiran C. Patel College of Osteopathic Medicine, Tampa, USA; 2 Internal Medicine, Moffitt Cancer Center, Tampa, USA; 3 Diagnostic Imaging and Interventional Radiology, Moffitt Cancer Center, Tampa, USA

**Keywords:** endovascular coil embolization, infective endocarditis, post-procedural bacteremia, ​streptococcus mutans, visceral artery pseudoaneurysm

## Abstract

Endovascular coil embolization is an effective, minimally invasive technique used to treat visceral pseudoaneurysms. Although rare, post-procedural infections such as bacteremia and infective endocarditis (IE) can occur, particularly in patients with underlying malignancy, valvular abnormalities, or immunosuppression. Early recognition and intervention are critical to preventing serious complications. A 52-year-old male with a history of renal cell carcinoma (post left nephrectomy) and a known heart murmur presented to the hospital with worsening left-sided abdominal pain. Imaging revealed multiple visceral pseudoaneurysms involving the hepatic, splenic, and mesenteric arteries. He underwent successful coil and glue embolization. Further workup with transesophageal echocardiography (TEE) revealed severe mitral regurgitation with vegetations and a flail posterior mitral leaflet. Blood cultures obtained after the embolization procedure were positive for *Streptococcus mutans*, confirming IE, and the patient subsequently underwent mitral valve replacement and pacemaker implantation at a cardiac hospital. He completed intravenous antibiotic therapy and was discharged in stable condition. Eighteen-month follow-up imaging showed evolving splenic infarcts without evidence of visceral pseudoaneurysms. This case highlights a rare but serious complication of coil embolization. While the causal link cannot be definitively confirmed, the sequence of events and known mechanisms of post-procedural bacteremia support a strong association. Factors such as the introduction of foreign material, ischemia-induced tissue vulnerability, and the presence of underlying malignancy may increase the risk of hematogenous bacterial seeding. *Streptococcus mutans*, though classically associated with dental flora, can exhibit invasive potential in vulnerable hosts. Cancer patients face elevated risks due to frequent vascular instrumentation and immunosuppressive treatments. High-risk patients may benefit from close post-procedural monitoring and early infectious workup. This case highlights the importance of species-level identification and clinical vigilance in preventing delayed diagnosis and complications.

## Introduction

A pseudoaneurysm occurs when blood collects outside an artery while remaining in communication with it. When comparing pseudoaneurysms to true aneurysms, pseudoaneurysms do not involve all the vessel wall layers, but they appear as they do. Some risk factors that predispose individuals to pseudoaneurysms include atherosclerosis, conditions that can cause increased blood flow, such as arteriovenous fistulas, hyperthyroidism, and trauma, and medial degeneration of the arterial wall. Coil embolization treats pseudoaneurysms by inserting a coil through a catheter to block blood flow and limit further expansion. This minimally invasive procedure prevents the pseudoaneurysm from rupturing and causing massive bleeding that may result in shock, organ failure, and even death [1]. Although coil embolization successfully repairs pseudoaneurysms, some complications may occur. An infrequent complication of coil embolization is bacteremia, which can lead to infective endocarditis (IE). IE is a life-threatening systemic condition characterized by infection of the endocardium and cardiac valves, commonly resulting in the formation of vegetations containing bacterial aggregations [2]. The introduction of bacteria during coil embolization poses a rare but serious risk, as transient or sustained bacteremia can lead to the colonization of cardiac valves, particularly in patients with predisposing factors such as pre-existing valvular disease or prosthetic valves. Once bacteria adhere to the endocardium, they trigger an inflammatory cascade, promoting fibrin deposition and vegetation formation, which can further propagate infection and embolic complications. Left untreated, this process may result in progressive valvular destruction, heart failure, and systemic embolization, underscoring the critical need for early detection and targeted antimicrobial therapy [3]. We present a rare case of IE as a complication following the repair of visceral pseudoaneurysms, highlighting the potential for severe systemic infection after endovascular interventions. 

## Case presentation

**Figure 1:**
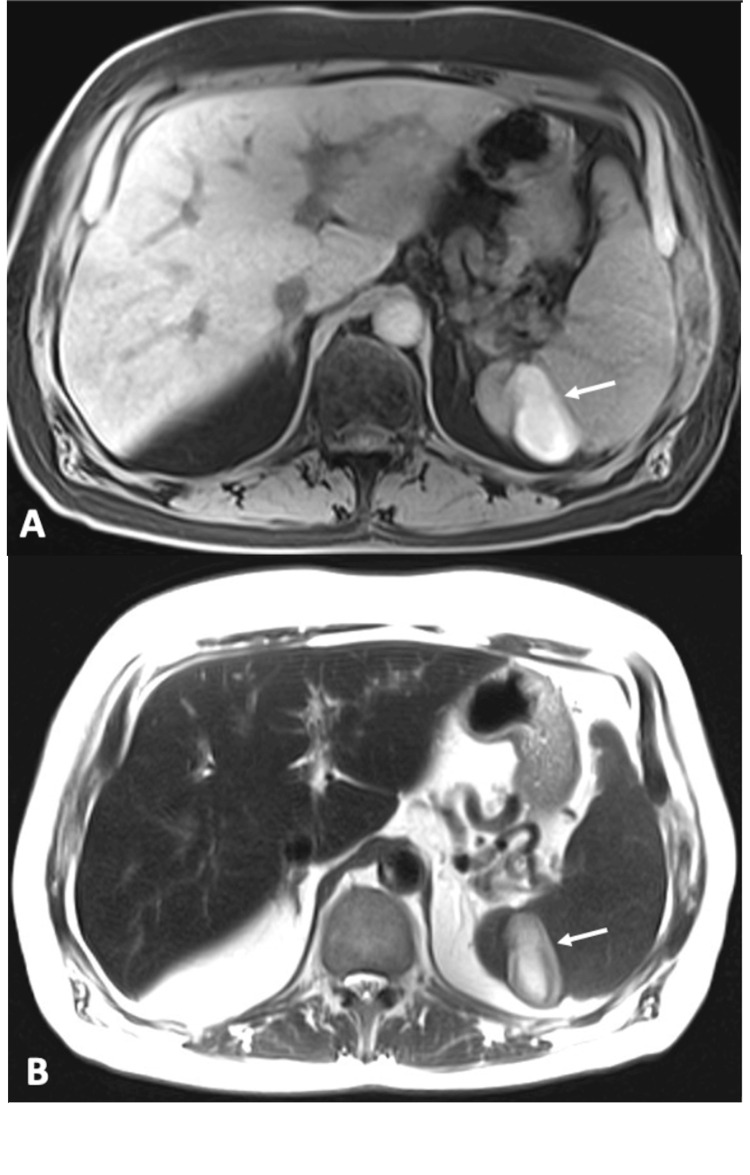
Axial T1-weighted fat-saturated (A) and axial T2-weighted (B) MR images demonstrated a 4.8 x 2.6 cm infarct in the spleen (arrows).

A 52-year-old male patient with a history of renal cell carcinoma, status post left nephrectomy, and a heart murmur presented to the hospital in June 2022 with a three-week history of abdominal pain. The pain radiated to his left flank and had worsened over the past three days. An outpatient abdominal MRI revealed a stable 1.4 cm lesion with nodular peripheral enhancement in the left hepatic segment and evolving splenic infarcts, which were new compared to a CT scan six months earlier (Figure 1). 

**Figure 2:**
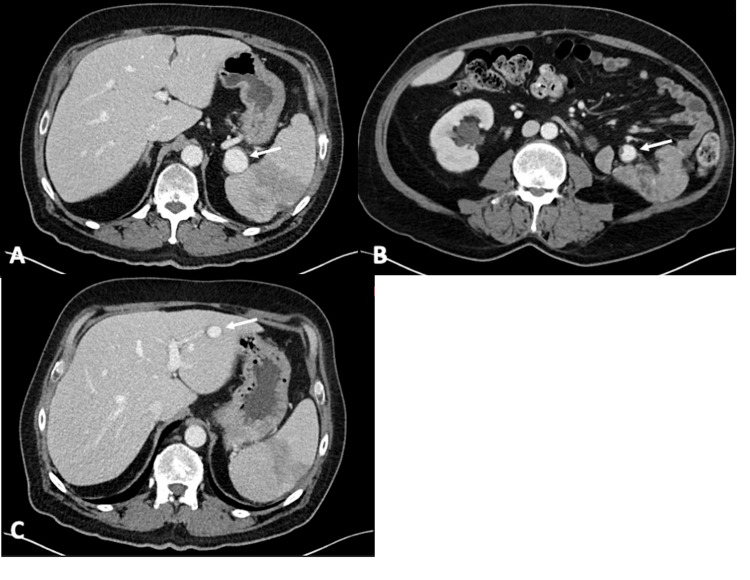
Axial contrast-enhanced CT images demonstrated a 2.7 cm pseudoaneurysm of the splenic artery (A), a 1.6 cm pseudoaneurysm in the small bowel mesentery (B), and a 1.5 cm pseudoaneurysm in the left lobe of the liver (C). Arrows point to the pseudoaneurysms.

The patient returned to the hospital three weeks later due to persistent abdominal pain. A CT scan demonstrated the development of rounded lesions adjacent to the spleen and in the small bowel mesentery, most consistent with pseudoaneurysms (Figures 2A, 2B). Additionally, the previously noted left hepatic lesion now showed a similar enhancement pattern, raising concern for a hepatic pseudoaneurysm (Figure 2C). On the following day, the patient underwent visceral angiography and successful coil embolization of the mesenteric and hepatic pseudoaneurysms and glue embolization of the splenic pseudoaneurysm. 

**Figure 3:**
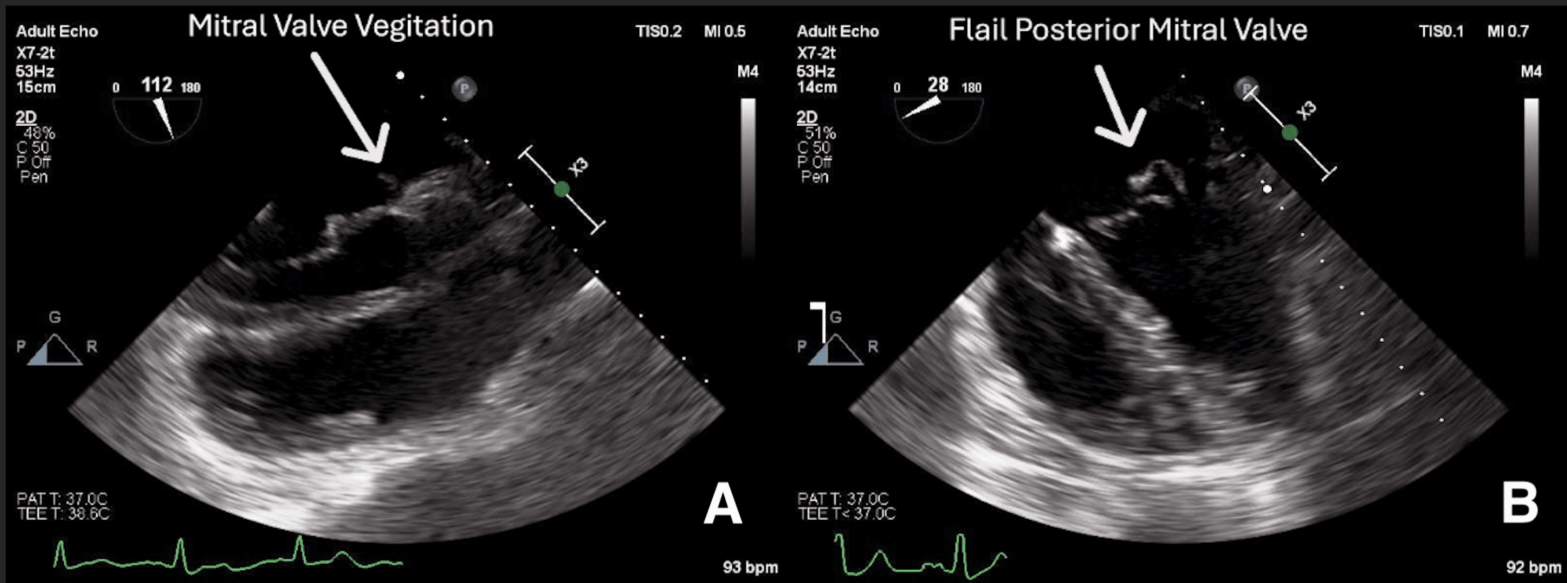
Transesophageal echocardiogram (TEE) demonstrated visible mobile vegetations attached to the mitral leaflet (A); TEE revealed a flail posterior mitral valve leaflet (B).

This clinical presentation raised suspicion for a septic embolic phenomenon as the underlying cause of visceral pseudoaneurysms and infarctions. Transesophageal echocardiogram (TEE) revealed severe mitral valve regurgitation with vegetations (Figure 3A), a flail posterior mitral leaflet (Figure 3B), and mild tricuspid regurgitation. Blood cultures were positive for *Streptococcus mutans*, confirming bacterial endocarditis. The patient denied any history of drug abuse and any history of recent dental procedures. The patient's dentition was unremarkable.

The patient was transferred to a cardiac hospital, where he underwent mitral valve replacement and pacemaker implantation. He received intravenous antibiotics, and apixaban was initiated for anticoagulation. His 54-day hospital course was also complicated by septic emboli leading to a stroke. 

**Figure 4:**
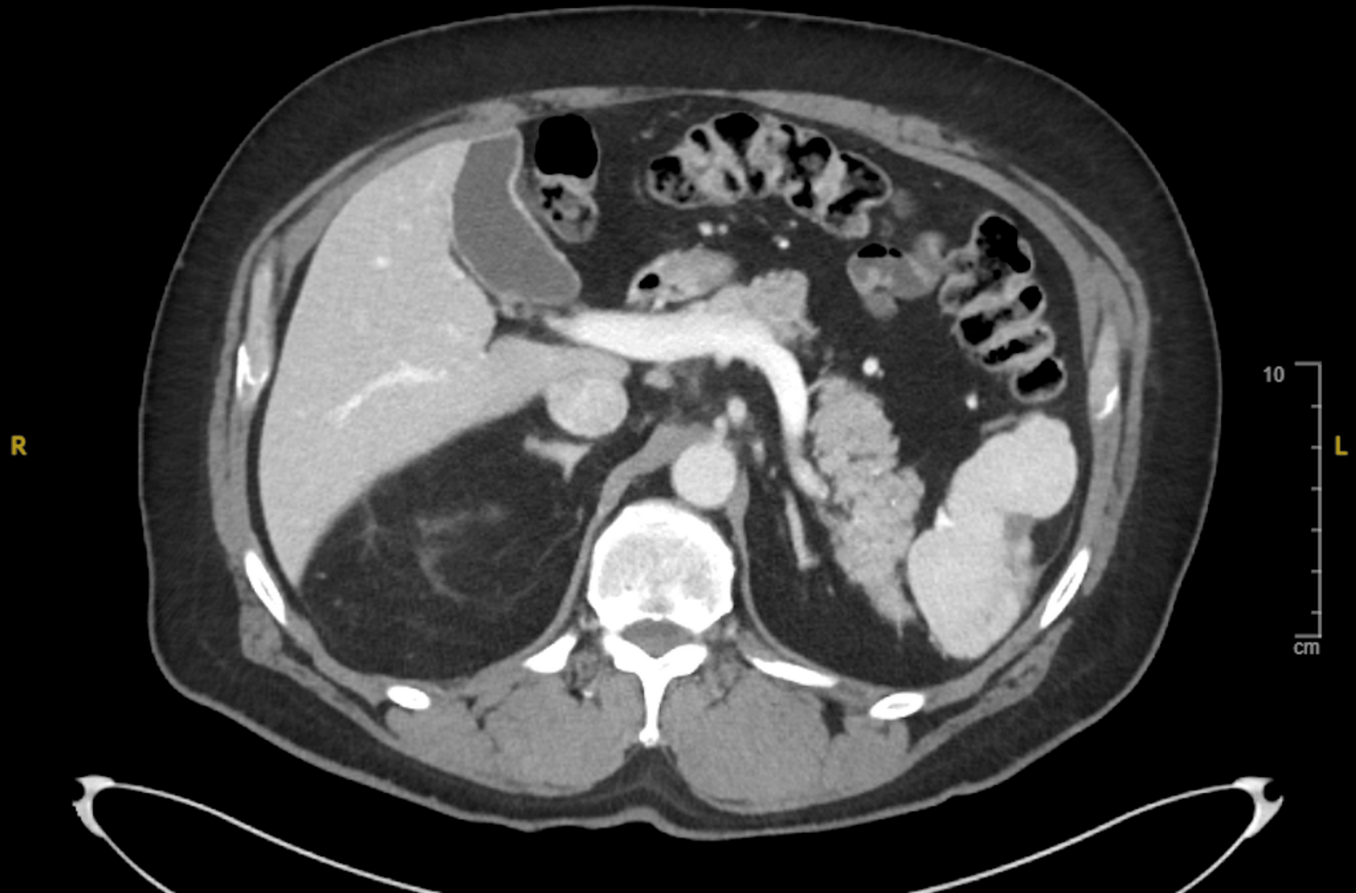
A CT scan of the abdomen with contrast demonstrated stable, evolving splenic infarcts without evidence of local tumor recurrence or new abdominal metastasis.

His latest CT imaging of the abdomen, performed in January 2025 (Figure 4), showed evolving splenic infarcts without evidence of visceral pseudoaneurysms or metastasis.

## Discussion

Endovascular embolization has become a cornerstone in the minimally invasive management of visceral and vascular pathologies. However, infectious complications, though rare, represent a significant clinical concern with potentially catastrophic outcomes. The presence of foreign material (such as coils or stents) inherently creates a nidus for bacterial adherence and biofilm formation, particularly when ischemic tissue or procedural contamination is present [4]. Infectious events have been documented across a spectrum of anatomical sites, ranging from intracranial abscesses to intraabdominal aortitis and graft infections [5,6]. Even more rare, however, is the development of IE, a complication observed in the present case following coil embolization. 

The pathogenesis of post-embolization infection may involve either direct contamination at the time of device placement or secondary hematogenous seeding from distant infections. In fact, transient bacteremia has been reported in up to 32% of patients undergoing angioplasty, though it is typically not associated with overt infection in most cases [7]. Inadequate aseptic technique, prolonged procedural times, or compromised sterile barriers in interventional suites have been cited as modifiable risk factors. Furthermore, coil embolization-induced ischemia and edema significantly exacerbate local tissue vulnerability, disrupting protective barriers and promoting bacterial invasion [8]. 

While *Streptococcus mutans* is historically associated with dental plaque and caries, emerging evidence highlights its potential as a significant pathogen in IE. Unlike many other viridians group streptococci (VGS) strains, *Streptococcus mutans* can express collagen-binding proteins (CBPs), such as the Cnm variant, which not only facilitate adhesion to damaged endothelium but may paradoxically inhibit platelet aggregation [9]. This dual action increases risk for both endocardial colonization and hemorrhagic complications such as intracerebral bleeding, especially when elastin-degrading metalloproteinases are co-expressed, contributing to vascular wall compromise [10]. These virulence factors may explain *Streptococcus mutans*’ association with not only IE but also hemorrhagic stroke in certain contexts. Dextran production by *Streptococcus mutans* and other VGS species like *Streptococcus sanguinis* and *Streptococcus mitis* has also been implicated in promoting adherence to endocardial surfaces, particularly following dental trauma [11]. Given its unique localization to the oral cavity and high affinity for collagen-rich tissues, minor mucosal trauma, such as flossing or dental cleaning, may precipitate transient bacteremia sufficient to seed vulnerable cardiac structures, especially in the presence of underlying valve pathology. 

Within the VGS taxonomy, *Streptococcus mitis* group strains are the most commonly isolated in IE, accounting for approximately 61% of culture-identified VGS cases, followed by *Streptococcus bovis* (15%), *Streptococcus mutans* (13%), and *Streptococcus anginosus* (9%) [12]. While *Streptococcus mutans* is a less frequent isolate in IE compared to *Streptococcus mitis*, it appears more disproportionately represented in IE versus all-cause bacteremia, suggesting a higher IE-to-bacteremia ratio [13]. Importantly, polymerase chain reaction (PCR) analysis of explanted valves has demonstrated superior sensitivity to culture in detecting VGS, further substantiating its role in endocardial colonization when blood cultures are inconclusive [12]. Recent epidemiologic data reinforce that species-level identification is critical in IE risk stratification. Bloodstream Infections (BSIs) caused by *Streptococcus mutans*, *Streptococcus sanguinis*, *Streptococcus gordonii*, and *Streptococcus gallolyticus* demonstrate high IE prevalence and should prompt echocardiographic evaluation irrespective of symptom severity [14,15]. In fact, multivariable analyses adjusting for patient-level risk factors have shown that infecting species remain independent predictors of IE risk, arguing against the umbrella use of “VGS” in clinical decision-making. The current consensus among experts now advocates for a tailored diagnostic approach that considers species-specific virulence, patient comorbidities, and prior valve pathology when evaluating streptococcal BSIs. 

Patients with underlying malignancy represent an important at-risk population for developing IE, particularly when coexisting factors such as immunosuppression, mucosal barrier disruption, and frequent exposure to invasive procedures are present. Immunosuppressive states may be attributed to the malignancy itself or to intensive treatment regimens such as chemotherapy and radiation [16]. Large-scale cohort data have shown increased incidence of IE among patients with colorectal, lung, or prostate cancers [17], and outcomes in this population are notably worse due to higher complication rates and a lower likelihood of receiving surgical intervention [18]. In addition to classic risk factors, many cancer patients undergo non-dental interventions, such as intravenous catheter placement, endoscopic procedures, or urologic instrumentation, that may serve as portals of entry for transient bacteremia and subsequent endocardial seeding [19]. Catheter-associated bacteremia remains a major contributor to IE in this population, with nearly one-quarter of cases directly attributable to long-term vascular access devices [20]. In the context of embolization procedures in oncology patients, this immunocompromised state may further compound the risk of post-procedural bacteremia and rare complications such as IE, underscoring the importance of heightened vigilance in this vulnerable cohort. 

Given the presence of multiple risk factors, such as the placement of foreign material, immunocompromised state, and the potential for virulent pathogens such as *Streptococcus mutans*, clinicians should maintain a low threshold in ordering a TEE in patients with several risk factors and signs of early systemic infections. Ordering a TEE can help guide prompt diagnosis of IE, guide antimicrobial therapy, and assist with preventing downstream complications such as embolic stroke. 

## Conclusions

This case highlights the rare but significant complication of IE following coil embolization, emphasizing the importance of heightened clinical awareness of post-procedural infections, particularly in high-risk populations such as patients with malignancy. Although direct causality between the embolization and subsequent IE cannot be definitively established, the association is supported by well-established pathophysiologic mechanisms and the temporal proximity of events. This patient exhibited multiple risk factors that collectively increase susceptibility to hematogenous seeding of bacteria, including the introduction of foreign material, ischemia-induced tissue damage, and an immunocompromised status. Our case further illustrates the pathogenic potential of *Streptococcus mutans*, an oral commensal with specific virulence traits that may facilitate endocardial colonization. As our understanding of species-specific risk within VGS evolves, and as post-embolization infectious complications remain under-recognized, this report reinforces the need for species-level microbiologic identification and close post-procedural monitoring. In vulnerable populations, including those with cancer, clinicians should maintain a low threshold for initiating a diagnostic workup for IE, especially the inclusion of a TEE, when signs of systemic infection or positive blood cultures with high-risk organisms are present. In addition, timely antibiotic therapy should be prompted to avoid delayed recognition and to improve clinical outcomes. 
